# Development of a Facemask System for Measuring Enteric Methane and Carbon Dioxide Production in Lactating Cows

**DOI:** 10.3390/ani16091365

**Published:** 2026-04-29

**Authors:** Beomseok Bae, Jungeun Kim, Chanhee Lee

**Affiliations:** Department of Animal Sciences, The Ohio State University, Wooster, OH 44691, USA; bae.303@osu.edu (B.B.); kim.9027@osu.edu (J.K.)

**Keywords:** greenhouse gases, GreenFeed, recovery, ruminants

## Abstract

The objective of this study was to develop a facemask system and evaluate its potential for measuring enteric CH_4_ and CO_2_ production. Recovery tests were conducted and resulted in 99.0% (SD, 3.2) and 102.3% (SD, 2.6), respectively. An experiment was conducted and the facemask system was compared with the GF system. Five mid-lactation cows were fed a lactation diet ad libitum (Period 1) for 5 days and then fed the same diet at 90% of the ad libitum intake for 5 days (Period 2) where CH_4_ and CO_2_ productions were measured at the end of each period. While daily CH_4_ and CO_2_ productions were different (*p* ≤ 0.04) between Period 1 and 2, those did not differ between the methods, and no interaction between period and method was observed. A linear regression analysis indicated a good correlation for CH_4_ (CCC = 0.72; RMSE = 22% of the mean) and CO_2_ (CCC = 0.79; RMSE = 10% of the mean) between the facemask and the GF system, while systemic bias was observed for CO_2_. In conclusion, the facemask system developed has the potential to measure CH_4_ and CO_2_ production from lactating cows and the measurement was similar with that from the GF system.

## Simple Summary

To quantify enteric CH_4_ emissions from ruminants, reliable measurement methods are essential. In this study, a cost-effective and user-friendly facemask system was developed to measure enteric CH_4_ and CO_2_ production, and its reliability was evaluated. The system primarily consisted of a mask, air filter, flowmeter, and air pump. During recovery tests, the facemask system recovered nearly 100% of CH_4_ and CO_2_ following the injection of known quantities of these gases into the mask. In an experiment to optimize breath sampling frequency, at least six time points, representing every 4 h sampling within a 24 h period, were sufficient to estimate daily CH_4_ and CO_2_ production. When compared with the GreenFeed system, a widely used method for measuring CH_4_ emissions in ruminants, the facemask system showed a strong correlation for both CH_4_ and CO_2_ production. These findings suggest that the facemask system has strong potential as a cost-effective, user-friendly, and reliable method for measuring enteric CH_4_ emissions in ruminants.

## 1. Introduction

The global livestock industry is estimated to account for 14.5% of total anthropogenic greenhouse gas emissions and livestock enteric methane (CH_4_) emissions are projected to account for 30.4% of global anthropogenic CH_4_ emissions in 2030 under the current emission trend [1,2]. With growing interest in global climate change, studies on enteric CH_4_ mitigation in ruminants have increased dramatically [3]. In such studies, reliable methods to measure enteric CH_4_ emissions from ruminants are essential to develop and evaluate CH_4_-mitigating strategies. A few in vivo techniques are currently available in measuring enteric CH_4_ emissions from ruminants, for example, the respiration chamber system, the sulfur hexafluoride (SF_6_) tracer technique, and the GreenFeed (GF; C-Lock Inc.) system [4,5]. Those existing methods have their own advantages and disadvantages. The respiration chamber system has been considered the gold standard and assumed to provide accurate CH_4_ measurement from individual animals, but it requires high construction and maintenance costs with limited throughputs [6]. The SF_6_ technique is relatively inexpensive, but its accuracy has been challenged and appears to be affected by many factors, such as inconsistent release of the tracer gas and equipment failure [5]. The GF system has advantages in its ease of use, suitability for large-scale experiments, and broad applicability in commercial settings, but the intake of the bait feed and cows’ visit patterns can affect its accuracy [5,7].

A facemask system has been widely used as an indirect calorimetry method to measure respiration and energy expenditure by quantifying exhaled gases from humans or animals in the sports and medicine fields [8]. In ruminants, the facemask system was first used in the early 20th century to quantify CO_2_ and CH_4_ production for the purpose of exploring energy metabolism [9]. The principle of the facemask system is similar to that of the respiration chamber or the GF system [6]. For example, the system operates with a one-way flow which sweeps entire gases exhaled from an animal through a facemask. The air containing exhaled gases passes the system during which the flow is measured, and the air is sampled and analyzed [6,10].

Despite its early development and the advantages, the facemask system has not been widely used in animal science research, probably due to the following limitations. The facemask system does not allow animals to eat feeds or drink water during the measurement, and therefore the measurement should only be used for a short period of time [5]. Oss et al. [11] observed that although a strong correlation (R^2^ = 0.72; *p* < 0.01) between the facemask system and the respiration chamber for CH_4_ production measured from 17 dairy bulls, CH_4_ yield (g/kg of DMI) measured by the facemask system had greater day-to-day (21.3 vs. 12.9%) and animal-to-animal (13.4 vs. 7.5%) variation compared with the respiration chamber. In that study, breath samples were collected at 6 h after feeding for 30 min over 3 days to measure CH_4_ production using the facemask system, while the respiratory chamber measured CH_4_ production every 15 min over 2 days. Similarly, Silveira et al. [12] observed no difference in CH_4_ and CO_2_ production between the facemask system (30 min breath sample collection at 4 h after feeding on 2 consecutive days) and the respiration chamber (every 20 min over 2 days) in 32 lactating cows. However, the authors concluded that the CH_4_ measurement using the facemask system needs to account for the diurnal variation in CH_4_ production to improve its accuracy according to the linear pattern of bias observed in the residual plot (CH_4_ from the respiratory chamber and CH_4_ from the facemask system). Probably because of relatively poor accuracy and high variability, only nine studies employed a facemask system for CH_4_ measurement in ruminants between 1994 and 2018, representing 2% of total published studies on CH_4_ measurement in ruminants during that period [13]. However, this limitation can be overcome using a spot sampling procedure which collects multiple samples with an equal sampling interval within the feeding cycle, thereby enabling coverage of the diurnal pattern of CH_4_ production [14,15].

The objectives of this study were to develop a facemask system that is cost-effective, user-friendly, and reliable in measuring enteric CH_4_ and CO_2_ production to optimize the spot breath collection procedure and validate measurement of daily CO_2_ and CH_4_ production in comparison with the GF system. We hypothesized that daily enteric CO_2_ and CH_4_ production measured by the facemask system using the optimized procedure would be comparable to those measured by GF.

## 2. Materials and Methods

All procedures involving animals in this study were approved by the Ohio State University Institution of Animal Care and Use Committee (IACUC: 2024A00000090).

### 2.1. Structure and Components of the Facemask System

**Figure 1:**
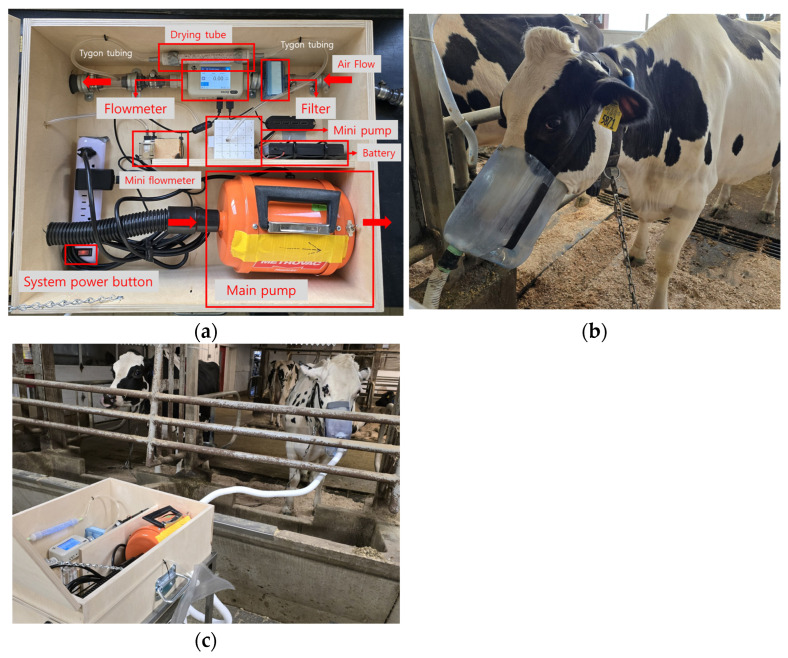
The facemask system developed to collect spot breath samples to determine daily CH_4_ and CO_2_ production from individual cows. (**a**) The structure and components of the facemask system. (**b**) A cow wearing the mask. (**c**) Collection of a breath sample using the facemask system.

The facemask system consisted of a mask, air filter, flowmeter, and air pump, which are connected via tubing (i.d. 3.2 cm; Figure 1a). The mask was made by modifying a 2.5-gallon water dispenser (2.5 Gallon H_2_O Oasis Beverage Dispenser, Arrow Home Products, Elk Grove Village, IL, USA) which is made of a flexible plastic material and allowed the mask to fit well on cows with different head sizes (Figure 1b). An air filter (5300-IF-LP, TSI Inc., Shoreview, MN, USA) is located before the flowmeter to remove potential particles in air flowing from the mask. An air flowmeter (Gas Flow Multi-Meter 5300, TSI Inc.) is positioned after the air filter and measures the air flow, temperature, and pressure and provides real-time flows (vol/min) at the standard temperature and pressure (STP; 0 °C, 101.3 kPa). The flowmeter is connected to the main pump (MagicAir^®^ Deluxe Inflator/Deflator Pump DIDA-4, MetroVac Vacuum Cleaner Co., Oakland, NJ, USA) via tubing, which creates a one-way flow of air (250 L/min) from the mask to the main pump. A Tygon tubing (i.d. 3.2 mm) is connected to the main flow tubing after the flowmeter. The other end of the Tygon tubing is connected to a battery-operating mini pump which pulls air samples from the air flow in the system. The mini pump continuously subsamples the air at 0.6 L/min (VFA-22-BV flowmeter, Dwyer Instruments, LLC., Michigan City, IN, USA) into a sampling bag (3 L Tedlar Sampling Bag 22501, Restek Co., Bellefonte, PA, USA.). The air samples collected pass through a drying tube (Drierite 26931 drying tube, W.A. Hammond Drierite Co., Ltd., Xenia, OH, USA), and dried air samples are collected in the sampling bag.

### 2.2. Facemask System Operation and Calculation of Daily CH_4_ and CO_2_ Production

To determine daily CH_4_ and CO_2_ production, the mask is positioned around the muzzle of a cow and gently tightened by a strap (Figure 1b). By turning on the main pump, all the exhaled breaths are collected into the system. When the flow is stable and the cow becomes calm, the mini pump is turned on and collects the air samples for 3 min from the main system into the sampling bag. According to the standardized spot breath sampling protocol, 3–7 min is required for a head–chamber system [7,16]. As a preliminary test, we evaluated 3, 4, and 5 min for breath sample collection internally using 3 lactating cows and observed no differences in CH_4_ and CO_2_ productions among these durations. To minimize stress and reduce restriction on normal behaviors (e.g., eating and drinking) during measurements with the facemask system, a 3 min sampling duration was selected for further evaluation. Immediately after or before the breath sampling, a barn air sample is also collected to determine the background levels of CH_4_ and CO_2_. The collected gas samples (1 bag per cow at each timepoint) are transported to the laboratory and analyzed for CH_4_ and CO_2_ concentrations using a photoacoustic multi-gas analyzer (Gasera One, Gasera Ltd., Turku, Finland). The background sample is also analyzed for CH_4_ and CO_2_ concentrations. For each timepoint, daily productions of CH_4_ and CO_2_ are calculated using the following equation (CH_4_ as an example):

Daily CH_4_ production (g/d) = Air flow (L/min at STP) × (CH_4_ concentration in the sample − background CH_4_ concentration) × CH_4_ density (g/L at STP) × 1440

We realized that acclimatization of all cows to the facemask system was necessary before experimental measurements to minimize stress-related behavior and ensure reliable breath collection. This is because a few cows were visually monitored for signs of discomfort or abnormal behavior, including head swinging, repeated attempts to remove the mask, excessive movement, or unstable breathing. Breath sampling was initiated only after the cow remained calm and airflow through the system was stable in the current study, and we did not observe abnormal behavior of cows during the experimental measurements in the current study. If abnormal behavior occurs during sampling, we recommend that the measurement is repeated or excluded from analysis. These precautions are necessary to reduce potential effects of animal behavior on respiration rate, exhaled breath collection, and estimated gas production.

### 2.3. A Recovery Test

A recovery test was conducted for CH_4_ and CO_2_ by introducing 200 mL of a standard gas (70% CH_4_ and 28% CO_2_) into the mask using a syringe for about 15 s while the facemask system was in operation without presence of animals. The air sample collected in the bag was analyzed and calculated for total volume of CH_4_ or CO_2_. The recovery was determined by dividing the measured volume of CH_4_ and CO_2_ by CH_4_ and CO_2_ injected into the mask, respectively. This recovery test was repeated 5 times. For CO_2_, an additional recovery test was conducted. During air flow sampling for 2 min, pure CO_2_ from a CO_2_ cylinder (Pre-filled 90-Gram CO_2_ Tanks, Air Venturi, Cleveland, OH, USA) was released into the mask. Before and after releasing CO_2_, the cylinder was weighed. Therefore, the recovery of CO_2_ was determined by dividing the measured amount of CO_2_ by the mass loss of the cylinder. This CO_2_ mass recovery test was conducted because this is a routine test for the GF system [14]. The CO_2_ mass recovery test was repeated 4 times.

### 2.4. Optimizing the Frequency of Spot Breath Collection: Experiment 1

To determine the optimal frequency of spot breath collection in a 24 h cycle (once daily feeding), 8 mid-lactation Holstein cows (average ± SD; 194 ± 21 DIM, 46 ± 5 kg/d milk yield, 687 ± 51 kg BW) were fed a mid-lactation diet (54% forage and 46% concentrate; 16% of CP, 30% of NDF, and 28% of starch on a DM basis) ad libitum and received the facemask system. Breath samples were collected from individual cows 16 times over 6 days to represent every 1 or 2 h sampling in a 24 h cycle. The breath sample was collected for 3 min at each timepoint. Daily CH_4_ production was determined at each timepoint point for individual cows, and all the measures (total 16 timepoints per cow) were averaged for each cow. Among those timepoints for each cow, the timepoints that represent every 3 h sampling (8 timepoints per cow), 4 h sampling (6 timepoints per cow), or 6 h sampling (4 timepoints per cow) in a feeding cycle were selected and averaged.

### 2.5. Comparison of CH_4_ and CO_2_ Production Between the Facemask System and the GF System: Experiment 2

For the comparison between the facemask system and the GF system, 5 mid-lactation Holstein cows (average ± SD; 156 ± 69 DIM, 43 ± 6 kg/d milk yield, 696 ± 34 kg BW) were housed in tie-stalls and fed a mid-lactation diet (50% forage and 50% concentrate; 17.5% CP, 27.6% NDF, and 26.5% starch on a DM basis). All cows received the diet for 2 weeks before the beginning of the experiment. The experiment consisted of 2 periods, and each period lasted for 7 days and breath samples were collected using the facemask and the GF system during the last 3 days in each period. In Period 1, cows were fed the diet for ad libitum intake (target refusal, 5% of feeds offered). Period 2 follows Period 1, and cows were fed at 90% of the ad libitum intake (as-fed basis) in Period 2. Ad libitum feeding in Period 1 and restricting feeding in Period 2 were implemented to create a difference in CH_4_ and CO_2_ production for each cow, allowing us to assess the sensitivity of the facemask and GF systems in detecting these differences. The diet was provided once daily (0700) with free access to water, and cows were milked twice a day (0400 and 1600).

Spot breath collection was conducted 8 times over 3 days to represent every 3 h sampling in a 24 h cycle in each period [14,15]. The following sampling schedule was used: 0200, 1100, and 2000 h (d 1); 0500, 1400, and 2300 h (d 2); and 0800 and 1700 h (d 3). Before the measurement, cows were trained for both the facemask system and the GF system once. Training for the facemask and GF systems were conducted in the same procedure and duration as the actual measurement during the experiment. Further training was not necessary because all cows did not refuse the measurement and did not show abnormal behavior (e.g., head swing) during the training measurement. Details of the GF operation and measuring procedure were described in Hristov et al. [14] and Ma et al. [17]. The GF system was calibrated by the built-in auto-calibration system and the mass CO_2_ recovery. The auto-calibration was performed once a week using a span gas (3.64% CH_4_ and 11.68% CO_2_ in N_2_; Air Liquide company). The mass CO_2_ recovery was performed at the beginning of the experiment. The recovery of CH_4_ and CO_2_ were 100.5 and 101.4%, respectively, from the auto-calibration, and the recovery of CO_2_ was 101.0% from the mass CO_2_ recovery test.

At each timepoint, cows were moved (one at a time) and confined in a chute, where the measurements were conducted using both the facemask system and the GF system. For GF measurements, the unit was moved to the cow in the chute and the cow’s head was positioned inside the GF hood. Exhaled breath was then collected for 5 min per cow using the GF system. Cows were restrained in the chute during GF measurements to eliminate potential errors that may occur when cows voluntarily approach the unit in response to a bait feed. The cow head needs to be maintained inside the GF hood for minimum 5 min to obtain a reliable result according to the manufacturer. However, when cows voluntarily access the unit, some cows frequently withdraw their heads from the hood during the 5 min measurement, which prolongs the measurement period and requires additional deliveries of the bait feed to keep the head inside the hood, resulting in interruptions in continuous CH_4_ collection and variability in bait feed intake among cows. To prevent these issues, cows were restrained in a chute to allow continuous measurements for 5 min and were provided an equal amount (100 g) of bait feed among cows. The facemask system was applied to each cow immediately after the GF measurement, and a breath sample was collected for 3 min per cow at each timepoint.

Due to an unexpected technical issue with the GF system, the measurements at the last 2 timepoints in Period 2 were not conducted (0800 and 1700 on d 3). Therefore, a total of 14 measurements per cow (8 measurements in Period 1 and 6 measurements in Period 2) for each method or 70 measurements per method were obtained (14 measurements per cow and 5 cows).

### 2.6. Statististical Analyses

To evaluate the optimal frequency of spot breath collection (Experiment 1), data were analyzed using PROC MIXED of SAS (version 9.4, SAS institute Inc., Cary, NC, USA) with the sampling frequency (all, 8, 6, and 4 timepoints) as a fixed effect and the cow as a random effect:

Y_ij_ = μ + F_i_ + C_j_ + e_ij_

where Y_ij_ = the dependent variable, μ = overall mean, F_i_ = a fixed effect of sampling frequency (i = 1 to 4), C_j_ = a random effect of cow (j = 1 to 8), and e_ij_ = residual error.

To evaluate the correlation between the facemask system and the GF system (Experiment 2), the data for each cow were checked for outliers (studentized residuals ≥ 3) using PROC REG. As a result, 5 observations (2 observations at 8 am in Period 1 and 2 observations at 5 am and 1 observation at 8 pm in Period 2) were identified as outliers, and a total of 65 observations per method were used for statistical analysis for both CH_4_ and CO_2_. A linear regression analysis was performed using PROC MIXED, with GF values as the dependent variable and the facemask values as the independent variable. The cow was included as a random intercept. The output from PROC MIXED was then used in PROC PLM to test whether the regression slope differed from 1. In addition to the linear regression analysis, the concordance correlation coefficient (CCC) and the root mean squared error (RMSE) were calculated as

$$RMSE=\sqrt{\frac{\sum_{i=1}^{N}{\left({Predicted}_{i}-{Actual}_{i}\right)}^{2}}{N}}$$

to assess estimation accuracy [18].

Data of CH_4_ or CO_2_ production (8 timepoints in Period 1 and 6 timepoints in Period 2 for each cow) were averaged by cow and period to obtain the daily CH_4_ or CO_2_ production per cow, method, and period, and the data were analyzed using PROC MIXED to determine the sensitivity in detecting differences in CH_4_ or CO_2_ production between the facemask and GF system using the following model:

Y_ijk_ = μ + P_i_ + M_j_ + P_i_ × M_j_ + C_k_ + e_ijk_

where Y_ijk_ = the dependent variable, μ = overall mean, P_i_ = fixed effect of period (i = 1 to 2), M_j_ = fixed effect of measurement method (j = 1 to 2), and P_i_ × M_j_ = the interaction between period and method. C_k_ = a random effect of the cow (k = 1 to 5) and e_ijk_ = residual error. The period (subject = cow) was included as the repeated measure with compound symmetry for the variance–covariance structure selected based on the lowest AIC.

Furthermore, the average CH_4_ or CO_2_ production data were analyzed to determine the effect of the period within each method using the same model without the interaction term. All data were presented as LSM, and mean differences were considered significant when *p* < 0.05 and to show tendency when 0.05 ≤ *p* < 0.10.

## 3. Results and Discussion

The recovery of CH_4_ and CO_2_ was 99.0% (SD = 3.2) and 102.3% (SD = 2.6), respectively, when a standard gas with known concentrations of CH_4_ and CO_2_ was introduced into the facemask system (Table 1). In the CO_2_ mass recovery test, we obtained the same recovery as above (102.3%, SD = 1.2). The recovery test is critical in a gas exchange system to assure the measurement of gas concentration and air flow and to confirm that the system is free from leaks [19]. A previous review by Hammond et al. [7] also emphasized that a recovery test should be performed regardless of the method chosen for CH_4_ measurement to identify potential sources of experimental error and minimize this error. If recovery is significantly different from 100% or appears to vary, it indicates that the experimental error exists and the source and variation in the errors should be identified and corrected. McLean and Tobin suggested that simply applying a correction factor obtained from the recovery test to compensate for the incomplete recovery is not desirable because it may lead to an inappropriate interpretation of the results [19]. A significant error source in the system (e.g., leakage) cannot be fully compensated by the correction factor [19]. In the current study, the average recovery values for CH_4_ (99.0%) and CO_2_ (102.3%) were close to 100% in the facemask system, and we decided not to use those as correction factors for adjusting the measures of CH_4_ and CO_2_. This decision was based on the suggestion from McLean and Tobin [19], and when values were adjusted with the recoveries of CH_4_ and CO_2_, the changes in daily production of CH_4_ and CO_2_ were trivial. Therefore, the construction and setup of the facemask system were appropriate to collect CH_4_ and CO_2_ in the mask and quantify those gases from individual cows.

**Figure 2:**
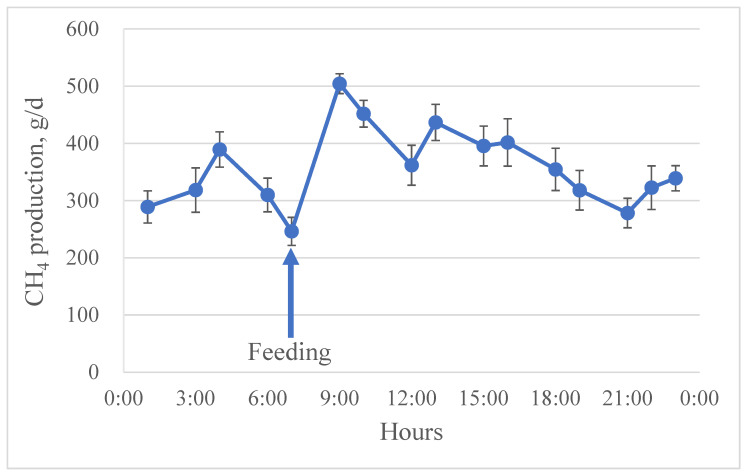
The diurnal pattern of CH4 production measured from lactating cows (n = 8) fed a diet once daily (07:00) using the facemask system. Error bars, SE.

In Experiment 1, we conducted a study to determine the optimal frequency of spot breath sampling using the facemask system. In previous studies, a facemask system was used to collect breath samples for 20 or 30 min [11,12]. Probably because of the relatively long measurement duration, individual animals received a single spot measurement on each day using the facemask system. However, a multiple spot sampling procedure has been widely adopted for the GF system [14]. Eight timepoints (every 3 h sampling) or six timepoints (every 4 h sampling) in a feeding cycle are likely sufficient to account for diurnal variation in CH_4_ production and to accurately estimate daily CH_4_ and CO_2_ production [14,15]. Indeed, daily CH_4_ productions from the GF system using the spot sampling procedure agreed strongly with those from the respiration chamber [17]. In the current study, the diurnal pattern of CH_4_ production was clearly observed using the facemask system as shown in Figure 2. The diurnal pattern of CH_4_ production agreed with previous studies where dramatic increases in CH_4_ production occurred after feeding and gradual decreases until the next feeding [20,21].

Results of CH_4_ and CO_2_ production obtained from the simulation evaluating various frequencies of spot breath sampling are shown in Table 2. Frequent spot breath sampling within a feeding cycle is required to account for diurnal variation in CH_4_ production, resulting in accurate estimation of daily CH_4_ and CO_2_ production for individual cows. According to previous studies, eight timepoints (every 3 h sampling) or six timepoints (every 4 h sampling) in a feeding cycle are likely sufficient to estimate daily CH_4_ and CO_2_ production [14,15]. However, those studies were conducted for the GF system or the respiratory chamber. In the current study, there was no difference in daily CH_4_ and CO_2_ production among the sampling frequencies (16, 8, 6, and 4 timepoints; Table 2). Despite no differences among the frequencies, the cow-to-cow variation for CH_4_ and CO_2_ production increased as the sampling frequency decreased. The results are in line with Lee et al. [15] where no difference in daily CH_4_ and CO_2_ production was observed among sampling frequencies from 24 to 4 timepoints in beef heifers and lactating cows while animal-to-animal variation increased as the frequency decreased. This suggests that as the sampling frequency decreases, the ability to detect differences in CH_4_ production among dietary treatments also decreases. Therefore, in that study by Lee et al., the authors recommended at least eight or six timepoints in a feeding cycle for spot breath sampling to estimate daily CH_4_ and CO_2_ production [15]. The cow-to-cow variations with the various sampling frequencies are highly comparable to the variations observed in Lee et al. [15], and we concluded that at least six timepoints in a feeding cycle were appropriate for spot breath sampling for the facemask system.

Daily CH_4_ and CO_2_ productions measured using the facemask system were compared with the GF system (Table 3). By the experimental design, DMI (25.2 vs. 29.8 kg/d; *p* < 0.01) and milk yield (39.8 vs. 43.9 kg/d; *p* < 0.01) were lower in Period 2 when cows were fed the diet at 90% of ad libitum intake compared with Period 1 when cows were fed ad libitum. The number of observations (five cows per treatment) was sufficient in power (alpha = 0.05, 1-beta > 0.80) to detect differences in DMI and milk yield by 10% in this experimental design [22], and we expected there was a true difference in CH_4_ and CO_2_ production between Period 1 and 2 due to the difference in DMI. When gas production data from the facemask and GF system were combined and analyzed, no interaction of period (ad libitum vs. restricted) by method (facemask vs. GF) was observed, indicating agreement between the two methods in detecting the changes in CH_4_ and CO_2_ productions that were intentionally induced between periods. While CH_4_ and CO_2_ productions did not differ between the methods, they were affected by period (dietary treatments). Cows in Period 1 (ad libitum feeding) had greater CH_4_ (418 vs. 384 g/d; *p* = 0.04) and CO_2_ production (16,162 vs. 14,834 g/d; *p* < 0.01) than those in Period 2 (restricted feeding). Because DMI is highly correlated with CH_4_ production in ruminants [23,24], the lower CH_4_ and CO_2_ productions in Period 2 were expected in the current study. When the data within each method were analyzed, we observed that CH_4_ production from the facemask system tended to be lower (375 vs. 416 g/d; *p* = 0.07) in Period 2 compared with Period 1 while the GF system did not detect a statistical difference in CH_4_ production between Period 1 and 2. For CO_2_ production, both methods detected a difference (*p* ≤ 0.02) between Period 1 and 2. The discrepancy between methods for detecting the change in CH_4_ production between Period 1 and 2 occurred due to the method-associated error (SEM for facemask and GF was 13.2 and 22.6, respectively). Despite the detection sensitivity difference, the analysis of the combined data indicates good agreement on CH_4_ and CO_2_ production between the facemask and the GF system (i.e., significant period effect with no interaction of period by method).

**Figure 3:**
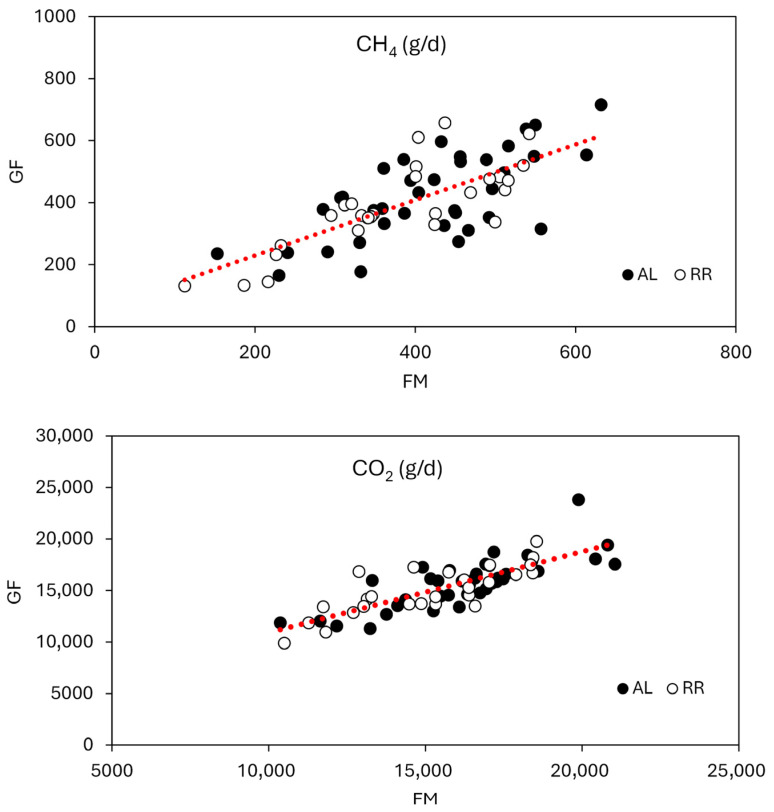
A linear relationship of CH_4_ and CO_2_ production measured between the GreenFeed system and the facemask system. FM = the facemask system; GF = GreenFeed; AL = ad libitum feeding in Period 1; RR = restricted feeding at 90% of AL in Period 2. CH_4_, GF = FM × 0.89 (SE = 0.102) + 52 (SE = 43.6). Y-intercept, *p* = 0.30. Slope (against 1), *p* = 0.28. RMSE = 93 (22% of the mean). CCC = 0.72. CO_2_, GF = FM × 0.81 (SE = 0.070) + 2692 (SE = 1150). Y-intercept, *p* = 0.08. Slope (against 1), *p* < 0.01. RMSE = 1561 (10% of the mean). CCC = 0.79.

This is supported by the results from the correlation analysis using a linear mixed model regression (Figure 3). We observed that the relationship was as follows: CH_4_ from GF (g/d) = CH_4_ from the facemask × 0.89 (SE = 0.102) + 52 (SE = 43.6), where the intercept (*p* = 0.30) and slope (*p* = 0.28) were not different from 0 and 1, respectively (CCC = 0.72; RMSE = 22% of the mean). For CO_2_ production, the relationship was as follows: CO_2_ from GF (g/d) = CO_2_ from the facemask × 0.81 (SE = 0.070) + 2692 (SE = 1150), where the intercept (*p* = 0.08) tended to be different from 0, and the slope (*p* < 0.01) was different from 1 (CCC = 0.79; RMSE = 10% of the mean). According to the slope, y-intercept, and CCC, the CH_4_ measurement was strongly correlated between the facemask and the GF system. For CO_2_, the slope was different from 1 and an intercept tended to differ from 0, indicating systematic bias. Although it is difficult to identify the sources, the systemic bias may have been from differences in procedures and standard CO_2_ gases used for calibration between the facemask and GF system. In addition, the procedure of measuring barn background CO_2_ concentration was different between the methods. For example, the GF system measured the background concentration before and after each measurement from cows while one background sample was collected after the breath collection of all cows was finished. Nevertheless, the CO_2_ measurement was also highly correlated between the facemask and the GF system within the current measurement range according to CCC and RMSE.

A few studies are available where a facemask system was compared with the respiration chamber system [11,12]. In those studies, however, a single spot sampling was conducted during a feeding cycle for the facemask system which failed to cover the diurnal variation in CH_4_ production and resulted in increased variability. Furthermore, the ability of the facemask system to detect differences in gas production among dietary treatments was not evaluated. Instead, such evaluation was conducted for the GF system, and its measurements were compared with the respiratory chamber [17,25,26]. In those studies, good agreements in daily CH_4_ production between those methods were observed. A study by Ma et al. observed a good correlation for CH4 production between the GF system and the respiratory chamber (r = 0.84) [17]. However, a weak correlation (r = 0.31, *p* = 0.23) between DMI and CH_4_ production was observed when measured using the GF system, which was weaker compared with the DMI and CH_4_ correlation for the respiration chamber (r = 0.58, *p* = 0.02). The authors discussed that the weaker correlation between DMI and CH_4_ production observed with the GF system occurred, at least in part, due to larger variation in CH_4_ production (SD, 64.1 vs. 48.5) in GF data compared with the respiration chamber. A study by Bayat et al. also found that the GF system showed greater variation (CV, 16.3 vs. 12.4%) in daily CH_4_ production compared with the respiration chamber [25]. In the current study, CH_4_ and CO_2_ productions were measured using both the facemask and GF under identical experimental conditions. The mean values of CH_4_ and CO_2_ production were almost identical. However, according to the effects of period within the method, CH_4_ production was less variable (SEM, 13.2 vs. 22.6) and therefore more sensitive in detecting the difference (*p*-value, 0.07 vs. 0.28) between periods for the facemask system compared with the GF system. Although the SEM for CO_2_ production was greater (442 vs. 282 g/d) for the facemask system compared with the GF system, both methods were able to detect a statistical difference between periods. From Experiment 2, we concluded that the CH_4_ and CO_2_ productions measured using the facemask system were comparable to those measured using the GF system. However, these results should be interpreted with caution because of the small sample size (n = 5). The cost of the facemask system developed in the current study was much less than any other in vivo methods and was about 3200 USD without the instrument measuring CH_4_ and CO_2_ concentration in gas samples. The most expensive component of the facemask system was the flowmeter (2500 USD).

### Limitations and Future Research

Despite the comparable results in CH_4_ and CO_2_ production measured using the facemask system in the current study, its limitations still exist. In Experiment 2, only five cows were used to compare daily CH_4_ and CO_2_ production between the facemask and GF systems (10 observations per method) and to examine the linear relationship between the two methods (65 observations per method). Therefore, the results from Experiment 2 should be interpreted with caution, and further studies with a larger number of animals are warranted. In addition, the facemask system has limitations in its application. While the GF system can be used in various research conditions (e.g., tie stalls, free stalls, and grazing), the facemask system can only be used at restrained settings such as tie stalls and is not applicable at commercial farms. In addition, measurements can be influenced by cows’ behavior, requiring animal training. We observed abnormal behaviors (e.g., head swinging) when the mask was positioned on the muzzle of a few cows, which may affect the respiration rate and the number of exhaled breaths during the measurement. However, we observed that such behaviors diminished or were eliminated as cows became accustomed to the mask through once or twice training sessions. In the current study, however, we did not observe any abnormal behaviors in cows during the measurements after training. Lastly, the current study examined the facemask system on CH_4_ and CO_2_ production. Because the facemask system collects all exhaled breath from the cow, the facemask system has the potential to monitor other gas components, such as O_2_ and H_2_. However, the accuracy of those gas measurements needs to be validated in future studies to expand the measurement scope of the facemask system.

## 4. Conclusions

A facemask system was developed appropriately according to the recovery of CH_4_ and CO_2_ that were close to 100%, and the measured CH_4_ and CO_2_ productions were comparable to those measured using the GF system. The good correlations in CH_4_ and CO_2_ production between the facemask system and the GF system and the ability of the facemask system to detect the differences in CH_4_ and CO_2_ production between periods in Experiment 2 demonstrate its potential as an alternative method that can be used in in vivo studies to quantify daily enteric CH_4_ production in ruminants. However, limitations of the facemask system on measuring daily breath gas production still exist, and further improvements and validation are necessary to determine its applicability and reliability.

## Figures and Tables

**Table 1:** The recovery test for CH_4_ and CO_2_ (%) in the facemask system.

Replications	Gases		
	CH_4_^1^	CO_2_^1^	CO_2_^2^
1	101.9	105.4	103.3
2	93.7	103.9	100.7
3	100.7	102.3	103.0
4	100.8	98.5	102.3
5	97.5	102.3	-
Average	99.0	102.3	102.3
SD	3.20	2.61	1.15
CV, %	3.24	2.56	1.13

^1^ The recovery test by introducing a gas mixture with known concentrations of CH_4_ and CO_2_ into the mask of the facemask system. ^2^ The recovery test by introducing pure CO_2_ gas from a CO_2_ cylinder into the mask of the facemask system.

**Table 2:** Optimizing the frequency of spot breath collection in a feeding cycle for daily productions of CH_4_ and CO_2_ using the facemask system.

Items	Sampling Frequency ^1^				SEM	*p*-Value
	All	8	6	4		
Gas production, g/d						
CH_4_	360	370	361	363	22.0	0.900
CO_2_	17,905	18,075	18,005	17,769	596.5	0.656
Cow-to-cow variation						
CH_4_, g/d						
SE	19.6	21.4	22.0	24.6		
CV, %	15.4	16.3	17.2	19.2		
CO_2_, g/d						
SE	552.3	596.1	616.1	619.3		
CV, %	8.7	9.3	9.7	9.9		

^1^ All, a total of 16 breath samples were collected over 6 days, representing every 1 or 2 h sampling in a feeding cycle (See Figure 2); 8, 8 breath samples out of 16 were selected that represent every 3 h sampling in a feeding cycle; 6, 6 breath samples out of 16 were selected that represent every 4 h sampling in a feeding cycle; 4, 4 breath samples out of 16 were selected that represent every 6 h sampling in a feeding cycle.

**Table 3:** Daily CH_4_ and CO_2_ productions (g/d) measured using the GreenFeed system and the facemask system.

Items ^2^	Period ^1^		Method ^2^		SEM	*p*-Values ^3^		
	1	2	GF	FM		P	M	P × M
DMI, kg/d	29.8	25.2			0.58	<0.01		
Milk yield, kg/d	43.9	39.8			2.85	<0.01		
GF								
CH_4_, g/d	421	393			22.6	0.28		
CO_2_, g/d	15,750	14,871			282.3	0.02		
FM								
CH_4_, g/d	416	375			13.2	0.07		
CO_2_, g/d	16,574	14,798			442.0	<0.01		
Combined								
CH_4_, g/d	418	384	407	395	15.1	0.04	0.46	0.69
CO_2_, g/d	16,162	14,834	15,310	15,686	298.3	<0.01	0.25	0.18

^1^ 1 = ad libitum feeding during Period 1; 2 = restricted feeding at 90% of ad libitum intake during Period 2. ^2^ GF = the GreenFeed system; FM = the facemask system; Combined = combined data between GF and FM. ^3^ P = the effect of period; M = the effect of method; P × M = the interaction between period and method.

## Data Availability

The data presented in this study are available on request from the corresponding author.
